# Comparative Study of Placental Allografts with Distinct Layer Composition

**DOI:** 10.3390/ijms26073406

**Published:** 2025-04-05

**Authors:** Pragya Singh, Acarizia Easley, Karla Tapia Menchaca, Victor Fanniel, Raymond Gomez, Joanna Marquez, Shauna Hill

**Affiliations:** RegenTX Labs LLC, 3463 Magic Dr Ste 315, San Antonio, TX 78229, USA

**Keywords:** CAMPs, tissue engineering, biomaterials, placenta, allografts, intermediate layer, extracellular matrix

## Abstract

Human placental-derived allografts are biomaterials categorized as cellular, acellular, matrix-like products (CAMPs) that can serve as wound coverings due to placenta tissue’s innate barrier function. The placental membrane consists of three layers, the amnion, the intermediate layer (IL), and the chorion, each contributing distinct functional and biological properties. This study investigates how variations in layer composition influence the Extracellular Matrix (ECM) and growth factor profiles of placental allografts. We compared Dual Layer (amnion–amnion), Full Thickness (amnion–intermediate–chorion, FT), and a novel four-layer allograft configuration (amnion–intermediate–chorion–amnion, ACA). Histological analyses using hematoxylin and eosin (H&E) and Masson’s trichrome staining revealed distinct structural architecture among the three allografts, with FT and ACA exhibiting 4.9 times and 5.7 times greater thickness as compared with the Dual Layer, respectively. Compositional studies revealed different concentrations of key ECM components (collagen, elastin, proteoglycans, hyaluronic acid) and growth factors (ANG-2, EGF, PDGF-AA, VEGF) across allografts. The collagen concentration was two times higher in ACA as compared with the Dual Layer and FT. Additionally, FT and ACA demonstrated higher levels of growth factors and other ECM components, underscoring their biochemical diversity. These findings highlight the fact that the structural and biochemical properties of placental-derived allografts depend on their layer composition. This study underscores the importance of tailoring layer configurations that are optimized for clinical applications of CAMPs, enabling clinicians to select the most suitable grafts for clinical use, such as for wound management.

## 1. Introduction

The human placenta serves as a protective barrier for the developing fetus, shielding it from external environmental factors and potential infections. This role is facilitated by its extracellular matrix (ECM) and associated components, which provides structural integrity and a supportive biological environment for fetal growth and development [1,2]. These attributes have led to the development of placental-derived materials that are utilized as a physical barrier to protect wound sites from contamination [3]. Placental-derived allografts are widely used in wound care settings and are commonly processed as single- or multi-layer constructs. Similar to skin grafts and synthetic scaffolds, placental allografts are categorized as cellular, acellular, matrix-like products (CAMPs) due to their role in providing structural support for injured tissue. Despite their clinical adoption, the impact of specific layer composition on the biochemical makeup of these constructs remains poorly characterized.

The placenta is composed of three main layers—the amnion, the intermediate layer (IL), and the chorion [4,5], each contributing distinct structural and biochemical properties. The amnion, the innermost layer, is composed of an epithelium, a basement membrane, a compact layer, and a fibroblast layer. Its ECM is particularly rich in collagens I and III as well as other matrix-associated proteins [6]. The IL, located between the amnion and chorion, contains proteoglycans, glycoproteins, hyaluronic acid (HA), and collagen type III, and serves as a reservoir for additional ECM components [6,7,8]. It also contains naturally occurring growth factors such as ANG-2 (angiopoietin-2), EGF (epidermal growth factor), PDGF-AA (platelet-derived growth factor), and VEGF (vascular endothelial growth factor). These growth factors, intrinsic to the IL, play essential roles in fetal development, participating in cellular processes such as vascularization, proliferation, and tissue remodeling [9,10]. The chorion, the outermost layer, contains the reticular layer, basement membrane, and trophoblast layer, with a dense ECM composed of collagens I, III, IV, V, and VI, along with other structural components [4,6,11,12].

The unique composition of each placental layer contributes to the overall biological and mechanical properties of the derived allograft. However, many commercially available placental-derived allografts do not retain all three layers in their final forms due to the IL’s susceptibility to separation during processing [4]. As a result, some allografts consist of only amnion, others include both the amnion and chorion layers, and some feature additional amnion layers that form tri- or quad-layer allografts [13]. These construct variations can lead to differential retention of collagen, growth factors, and other ECM components [4,14], which can consequently affect the allograft’s utility as a wound covering that also serves as a physical barrier to protect the wound [6,15]. For example, collagen is essential for forming a protective barrier, providing mechanical strength and durability to placental allografts [10,16,17,18], while elastin enhances flexibility and resilience [19,20]. Proteoglycans and HA contribute to matrix organization and hydration, and the retention of growth factors may sustain the biological environment [21]. Despite compositional differences, many of these allografts report the presence of vital placental components after processing and dehydration, which may contribute to their wound-protective properties [13,22,23]. Although several allografts have demonstrated clinical success in supporting wound closure as well as standard of care [24,25,26], comparative studies of placental allografts that evaluate the impact of compositional layer designs remain scarce.

This study evaluated and compared the compositional makeups of three human placental-derived allografts with distinct layer compositions. The allografts included an established dual-amnion graft technology (Dual Layer); a full-thickness graft that comprised all three placental layers, including the IL (Full-Thickness, FT); and a novel full-thickness graft that featured an added amnion layer comprising a four-layer configuration (amnion–IL–chorion–amnion, ACA). This study shows the differences in collagens, elastins, proteoglycans, HA, and growth factor levels among these three allografts. These key findings may offer valuable insights into the optimization of CAMPs for their intended biomedical applications.

## 2. Results

### 2.1. Physical Characteristics of Dual Layer, Full-Thickness, and ACA

**Figure 1:**
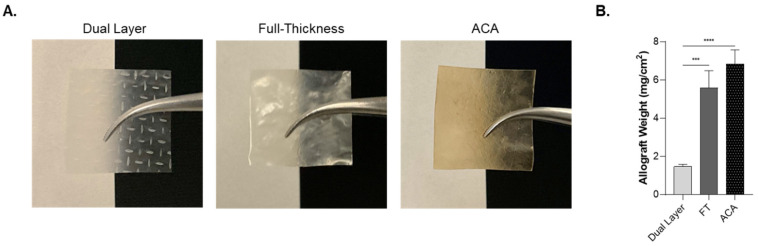
Structural and physical differences between each placental-derived allograft. (**A**) Representative images of Dual Layer, FT, and ACA. (**B**) The comparative weight analysis representing the mean weight of eight donors per CAMP technology, normalized to the surface area, shows the distinct compositions of FT and ACA as compared with Dual Layer. The error bars represent the standard error of the mean (SEM). *** *p* < 0.001; **** *p* < 0.0001.

This study compares three human placental membrane-derived allografts: Dual Layer, FT, and ACA, a novel four-layer allograft. Dual Layer is composed of two amnion layers; FT contains amnion, IL, and chorion; and ACA contains the same three layers of FT along with an additional amnion layer beneath the chorion. Visual evaluation of the allografts demonstrate that each allograft exhibited a distinct appearance, as shown in Figure 1A. Dual Layer is thin and translucent, while FT and ACA are visibly thicker and opaque. Additionally, ACA has a brown hue, distinguishing it from the other two allografts.

The weight of each allograft was measured and normalized to its surface area. Both FT and ACA weigh significantly more per unit area as compared with Dual Layer (Figure 1B). Additionally, comparative analysis of graft thickness was performed utilizing H&E (hematoxylin and eosin) micrographs. The overall mean thickness of FT was 4.9 times greater than Dual Layer, while ACA was 5.7 times greater, which is consistent with the weight measurements (Table 1).

### 2.2. Differences in Structural Architecture Between Dual Layer, Full-Thickness, and ACA

**Figure 2:**
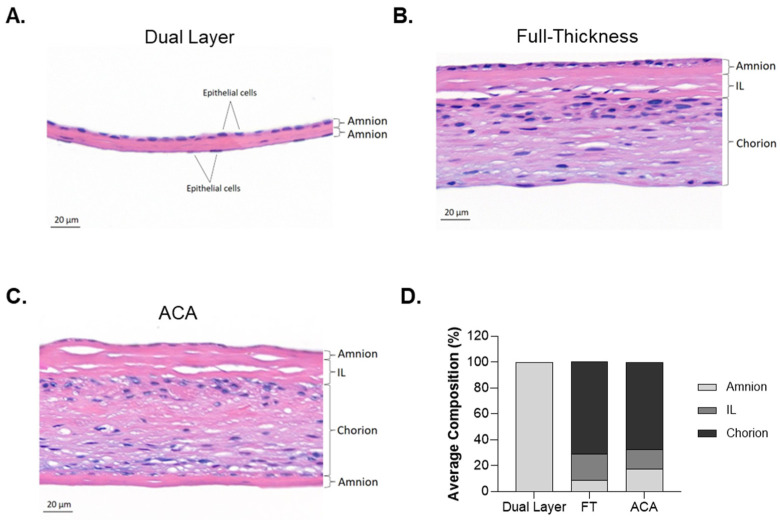
Distinct histological and structural composition of different placental-derived allografts. (**A**–**C**) Representative cross-sectional micrographs of Dual Layer, FT, and ACA allografts stained with H&E confirmed the presence and orientation of the placental layers in each allograft. Scale bar = 20 μm. (**D**) Stacked column graph illustrating the percentage compositional analysis of each layer in seven donors of each CAMP technology.

To validate the presence and orientation of the placental layers in each allograft, histological analysis was conducted using H&E staining. The staining confirmed the integrity of the layers and highlighted structural differences among the allografts (Figure 2). The Dual Layer is comprised of two distinct amnion layers, indicated by epithelial cells with purple-stained nuclei on the apical surfaces on both the upper and lower layers of the allograft (Figure 2A). This dual-amnion structure represents a simple bi-layered architecture composed of 100% amnion (Figure 2D). The FT allograft depicts all three major placental membrane layers (Figure 2B). The amnion layer is located at the top, followed by the IL and the chorion layer. The IL has a distinct acellular spongy structure, and the chorion presents a denser structure, where trophoblasts can be observed by the nuclei that are stained blue-purple. Quantitatively, the FT allograft is composed of approximately 9 ± 3% amnion, 20 ± 7% IL, and 71 ± 10% chorion (Figure 2D). The ACA has a similar structure to FT but includes an additional amnion layer beneath the chorion. The additional amnion layer is positioned with its stromal side neighboring the chorion layer and its epithelial side on the apical surface (Figure 2C). The layer composition of ACA consists of approximately 17 ± 7% amnion, 15 ± 10% IL, and 67 ± 17% chorion (Figure 2D). Despite the intrinsic donor variability of each allograft (Table 1), this comparative analysis highlights the structural differences among the allografts, with ACA showing a unique architecture compared with the other allografts due to its extra amnion layer.

### 2.3. Influence of Compositional Layering on Total Protein Content

**Figure 3:**
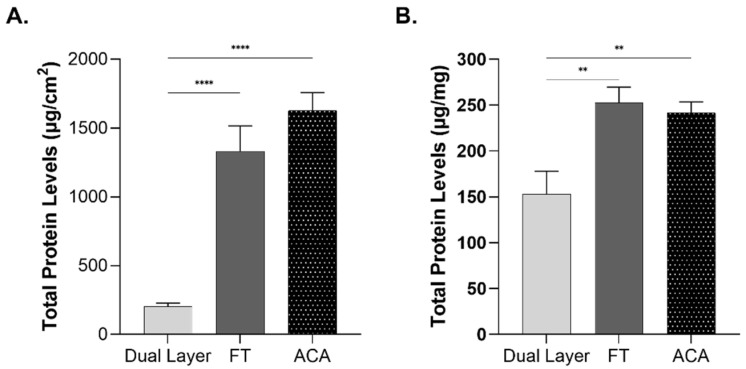
Increased total protein levels in FT and ACA. Mean total protein concentration was normalized (**A**) by surface area and (**B**) by weight in eight donors per CAMP technology, highlighting significantly higher protein levels in FT and ACA compared with Dual Layer (** *p* < 0.01; **** *p* < 0.0001). Error bars represent the SEM.

Protein content was measured in placental allografts to investigate whether variations in layer composition influence the overall protein levels. To ensure consistent comparison between the allografts, the total protein was normalized to both surface area and weight. Both FT and ACA exhibited significantly higher levels compared with Dual Layer (Figure 3A). FT demonstrated a 6-fold increase, while ACA showed a 7-fold increase relative to Dual Layer. These differences reflect the inclusion of additional structural layers in FT and ACA. When normalized to the overall allograft weight, FT and ACA still exhibited significantly higher levels of total protein concentration, containing approximately 1.6 times more compared with Dual Layer (Figure 3B). These findings underscore the impact of layer composition and thickness on the total protein content of placental-derived allografts.

### 2.4. Impact of Compositional Layering on ECM Content

**Figure 4:**
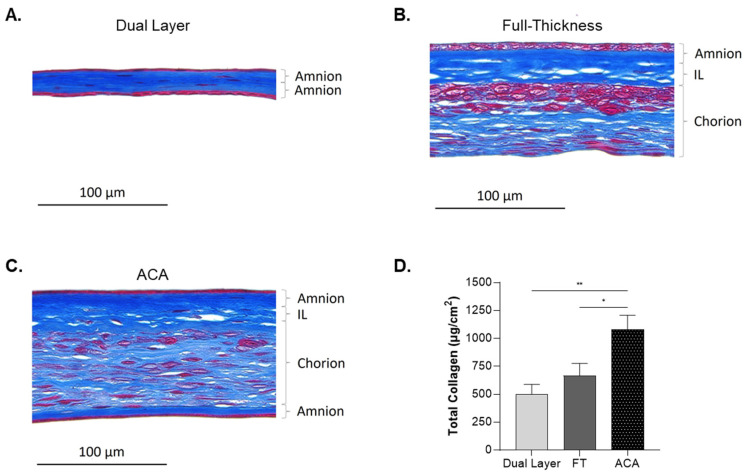
ACA exhibits higher levels of collagen compared with Dual Layer and FT. (**A**–**C**) Masson trichrome staining showed the presence of collagen in different patterns according to the type of allograft. Scale bar = 100 μm. Images were taken at 40× magnification to highlight the differences among allografts. IL, intermediate layer. (**D**) Quantification of total collagen showing higher levels in ACA compared with the other allografts (*, *p* < 0.05; **, *p* < 0.01). Levels are represented as the average of at least seven donors per CAMP technology. Error bars represent the SEM.

Collagens are a critical group of proteins in the ECM of the placental membrane [27]. Therefore, the distribution of collagen across the placental layers of each allograft was evaluated using Masson’s trichrome staining of tissue cross-sections. In Dual Layer, intense blue staining between the epithelial layers of amnion was observed, indicating a high density of collagen in its simple bi-layer structure (Figure 4A). This result highlights the fact that, despite its simplicity of construction, Dual Layer is rich in collagen. In FT, collagen was distributed across all three placental layers (Figure 4B). The amnion layer exhibited dark blue staining, followed by the IL, which showed a similarly dense collagen content. The chorion displayed lighter blue staining, reflecting a lower collagen density compared with the other layers. These findings demonstrate that collagen is present throughout FT but varies in staining across the layers, corresponding to its more complex structural architecture. In ACA, which incorporates an additional amnion layer, intense blue staining was observed in both the upper and lower amnion layers, as well as in the IL, while the chorion displayed a lighter blue stain (Figure 4C).

To verify Masson’s trichrome results, the quantitative levels of total collagen per surface area were determined and compared among each allograft (Figure 4D). ACA demonstrated a significant increase in the concentration of collagen compared with both Dual Layer and FT, with approximately double the amount. The results corroborate the intense blue staining observed in ACA during Masson’s trichrome staining (Figure 4C). In contrast, FT showed no significant differences in collagen content as compared with Dual Layer, though it trended higher (Figure 4D).

**Figure 5:**
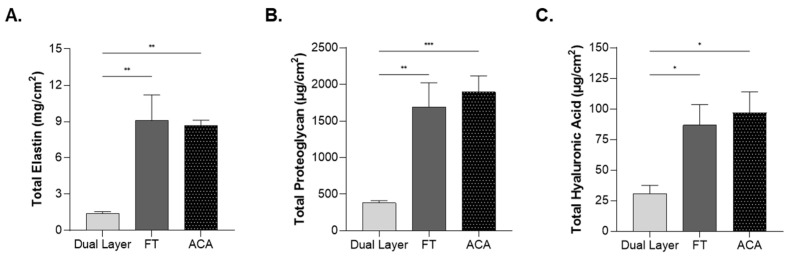
FT and ACA allografts contain higher levels of ECM components than Dual Layer. (**A**) Elastin, (**B**) proteoglycan levels, and (**C**) hyaluronic acid were quantified in at least seven donors per CAMP technology. Error bars represent the SEM. * *p* < 0.05; ** *p* < 0.01; *** *p* < 0.001.

The content of other relevant ECM proteins was also evaluated. Levels of elastin, proteoglycan, and HA were quantified and normalized to the surface area. This approach reflects the total amounts of these factors present in the allograft. Elastin was found to be significantly higher in FT and ACA as compared with Dual Layer (Figure 5A). Both FT and ACA exhibited approximately a 7-fold increase in elastin content as compared with Dual Layer. Proteoglycans were demonstrated to be significantly higher in FT and ACA as compared with Dual Layer, with both allografts demonstrating about a 2-fold increase (Figure 5B). HA was found to be significantly higher in both FT and ACA, showing a 2-fold increase in HA content as compared with Dual Layer (Figure 5C). These findings suggest that the IL and chorion may enhance the levels of proteoglycans and HA in these allografts.

### 2.5. Influence of Compositional Layering on Growth Factors Levels

**Figure 6:**
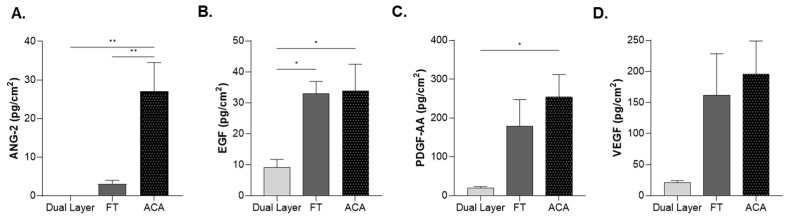
Growth factor levels vary among all three placental-derived allografts. Growth factors (**A**) ANG-2, (**B**) EGF, (**C**) PDGF-AA, and (**D**) VEGF were quantified in eight donors per CAMP technology. Error bars represent the SEM. * *p* < 0.05; ** *p* < 0.01.

The levels of several key growth factors, including ANG-2, EGF, PDGF-AA, and VEGF were quantified by enzyme-linked immunosorbent assay (ELISA) (Table 2, Figure 6). The data were normalized by surface area to compare the total amounts of each growth factor across the three allografts (Table 2). Levels of growth factors in all placental-derived allografts were quantified (Figure 6). ANG-2 levels in ACA were 8.9 times higher than those in FT. EGF levels were approximately 3.6 times higher in both FT and ACA as compared with Dual Layer. PDGF-AA levels were significantly elevated, with FT showing an 8-fold increase and ACA a 9-fold increase. VEGF levels increased by 7.8-fold in FT and 9.4-fold in ACA (Figure 6). Although VEGF levels were not statistically significant due to inherent sample variability, they remained consistent across samples. Together, these findings indicate a greater presence of growth factors in FT and ACA.

## 3. Discussion

This study is the first to directly compare the biochemical profiles of complex, novel placental allograft configurations with a traditional amnion-only allograft. We evaluated three placental-derived allografts: Dual Layer, which consists solely of amnion; FT, which comprises amnion, IL, and chorion; and ACA, a quad-layer configuration composed of amnion, IL, chorion, and the retainment of an additional amnion. Although previous studies have compared individual layers to tri-layer allograft constructs, none have evaluated advanced allografts side by side. Moreover, this is the first study to provide a detailed biochemical characterization of complex placental allografts by examining key ECM components such as collagen, elastin, proteoglycans, and HA, as well as native growth factors including ANG-2, EGF, PDGF-AA, and VEGF.

One of the most noteworthy findings was that FT and ACA contain significantly higher protein content levels compared with Dual Layer when normalized to bothr surface area and graft weight. Because ECM proteins are central to forming a cohesive barrier at injury sites, these findings suggest that retaining more placental layers yields an allograft with greater biochemical complexity [4,12]. This difference was reflected in the more opaque and thicker appearance of FT and ACA compared with the translucent Dual Layer, highlighting how additional layers may substantially affect the final allograft’s overall composition.

Collagen emerged as a prominent ECM component in ACA, which is notable given its recognized role in providing mechanical strength and structural integrity in the ECM [28]. Its supercoiled helical structure provides resistance to mechanical stress and contributes to tensile strength [29]. Studies have suggested that collagen content influences the mechanical properties of placental-derived allografts [4,8,13], and collagen-based barriers are associated with a reduced risk of external contamination while supporting local tissue integrity [30]. Additionally, previous research has demonstrated that tri-layer allografts, which preserve collagen-rich layers, exhibit greater tensile strength and slower degradation rates compared with single- and bi-layer allografts [13]. This enables the graft to remain intact over a longer period, providing sustained mechanical support and a stable barrier at the wound site. The prolonged presence can reduce the need for frequent reapplication and can lower the risk of contamination [13]. The rich collagen composition in ACA is expected to improve the allograft’s mechanical stability, resilience, and handling characteristics in clinical settings, making it a viable option for wound management.

In addition to collagen, elastin plays an important role in tissue flexibility and resilience, particularly in maintaining the biochemical properties of the ECM. Elastin was also found to be elevated in FT and ACA, which aligns with the presence of the chorion layer, which is known to be a primary source of elastin in placental membranes [15]. Elastin plays a role in tissue elasticity and mechanical resilience, contributing to the overall flexibility of allografts [4,31]. Studies have demonstrated that introduction of elastin onto collagen-based scaffolds enhances elasticity, improving the material’s overall ability to adapt to mechanical stress while maintaining structural integrity [32]. Others have analyzed the utility of elastin, with some suggesting that elastin may be an important protein for increasing the flexibility of tissue coverings [33,34]. This suggests that the increased elastin content in FT and ACA may contribute to greater flexibility, which may be beneficial if these allografts are used for other purposes, such as wound coverings.

Proteoglycans and HA were found in higher concentrations in FT and ACA as compared with Dual Layer. Proteoglycans and HA are known to facilitate hydration and structural organization in various tissues [35,36,37]. Their presence in FT and ACA complements the mechanical properties of collagen and elastin, highlighting the potential advantages of retaining the FT structure in placental-derived allografts. The greater abundance of ECM constituents in FT and ACA, combined with their thicknesses, may enhance overall strength and help form a strong, protective barrier at the wound site, particularly when compared with Dual Layer.

In addition to ECM constituents, this study revealed that intrinsic growth factors (ANG-2, EGF, PDGF-AA, and VEGF) were present at higher levels in FT and ACA as compared with Dual Layer. This aligns with previous reports that IL is a major source of these growth factors [4,38]. While our study did not directly evaluate the clinical outcomes, the role of these growth factors is well established [39,40,41]. For instance, ANG-2 is associated with angiogenic regulation; EGF is associated with cell proliferation, differentiation, and cell survival; PDGF-AA is associated with cell recruitment; and VEGF is associated with endothelial cell function [42,43,44,45,46,47,48]. Furthermore, prior studies have linked the presence of intrinsic growth factors with improved outcomes in wound management [6].

The presence of these growth factors in FT and ACA suggests that key biomolecules remain intact following processing, with profiles comparable to those of previously characterized dehydrated human amnion/chorion membranes (hDACMs) [6,22,23]. These biomolecules have been identified in wound healing environments and are associated with processes such as endothelial cell proliferation and angiogenesis in vitro and preclinical animal studies [6]. Additionally, the clinical utility of hDACMs has been demonstrated in multiple studies, in which their use in combination with standard of care yielded favorable results compared with standard of care alone [26] or to other skin substitutes [49]. The increased presence of these native growth factors in FT and ACA suggests a more complex biochemical environment compared with Dual Layer, which can be advantageous for wound covering applications by supporting a conducive microenvironment.

Altogether, the data suggest that retaining all three layers (FT) or preserving a secondary amnion layer in FT (ACA) may offer advantages over traditional amnion-only placental allografts by maintaining higher levels of ECM components and growth factors. In cases where a thicker covering with a broader biochemical profile is needed, FT and ACA might be favored. These findings underscore that processing decisions regarding which placental layers to retain directly affect the allograft’s biological properties. Future studies should investigate how these compositional differences affect placental allografts’ abilities to support wound management and correlated specific layer configurations with clinical outcomes. Bridging this gap will be key for refining allograft design and expanding its potential utility in clinical settings.

## 4. Materials and Methods

### 4.1. Preparation of Placental Allograft

The placentas were acquired at the time of delivery from healthy birth mothers of 18 years and older, of all races, with tissues obtained from both cesarean sections and vaginal deliveries. All donors were screened and tested in accordance with U.S. Food and Drug Administration (FDA) regulations and the American Association of Tissue Banks (AATB) standards to ensure the safety and quality of the tissue. Allografts were generated from donated placentas using processing techniques characterized as minimally manipulative. Blunt dissection was used to separate the membrane from the placental disc, followed by rinsing with buffered salt and detergent solutions.

FT retained the natural amnion–IL–chorion structure. ACA, derived from FT, preserves a secondary delaminated amnion layer during processing, which naturally adheres due to stromal surface properties. Dual Layer was created by delaminating the amnion from the chorion and folding it onto itself, with the stromal side for both layers facing inward. All allografts underwent controlled drying and were heat-sealed in packaging without the use of air or inert gas. Sterilization was performed using low-dose gamma-irradiation in compliance with ISO-11137 [50].

### 4.2. Histological Analysis

Dual layer, FT, and ACA allografts were fixed in 10% neutral-buffer formalin overnight at 4 °C. The 5 μm thick cross-sections were stained for histological analysis by the Precision Pathology Laboratory (San Antonio, TX, USA) according to the standard procedures. H&E staining was selected to evaluate the general overview of the placental tissue structure and Masson’s trichrome staining was selected to assess the collagen content and structural integrity of the allografts. The representative cross-section images of H&E were taken using a Slide Viewer 2.8.0, and Masson’s trichome was taken using a BioTek Cytation 5 Multimode Reader (Agilent Technologies, Santa Clara, CA, USA) at 40× magnification. For all histological analyses, seven distinct donors were evaluated for each CAMP technology.

### 4.3. Compositional Layer Analysis

Compositional layer analyses of Dual Layer, FT, and ACA allografts were performed using H&E-stained images analyzed in SlideViewer 2.8.0. Four representative areas of the cross-sections were selected for the measurements of the amnion, IL, and chorion across the samples. Results were presented as the mean thickness measured in micrometer units of each layer across the different CAMP technologies ± their respective standard deviation (SD). Proportional layer composition was calculated as the thickness of each layer relative to the total thickness of the allografts. Results were reported as the average of percentages ± SD. For all placental layer compositional analyses, seven distinct donors were evaluated for each CAMP technology.

### 4.4. Quantification of Extracellular Matrix Components

Biochemical analyses of the placental allografts, cut to a standard size of 0.25 cm^2^, were conducted to quantify the ECM using a CLARIOstar microplate reader (CLARIOstar, BMG Labtech, Ortenberg, Germany). The concentrations were determined by absorbance, and the standard curves generated from each biochemical assay were normalized to the graft surface area. For all ECM studies, at least six distinct donors were evaluated in technical triplicates across two independent experiments per CAMPs technologies. Results were normalized to the graft’s surface area.

#### 4.4.1. Collagen

Collagen levels were quantified using the QuickZyme Total Collagen Assay kit (QuickZyme, Leiden, The Netherlands, Cat. No. QZBtocol2) according to the manufacturer’s instructions. Briefly, allografts were digested in 1 mL of 6 M Hydrochloric Acid (HCl) at 95 °C for 20 h. After centrifugation, the supernatant was diluted to a final concentration of 4 M HCl and transferred to a 96-well microplate. Samples were further diluted 5× with 4 M HCl and incubated with assay buffer, followed by an incubation with detection reagent. The levels of total collagen were quantified by absorbance at 570 nanometers (nm).

#### 4.4.2. Elastin

Elastin levels in the allografts were measured using a fastin–elastin assay (Biocolor, Carrickfergus, UK, Cat. No. F4000), following the manufacturer’s instructions with slight modifications. In brief, allograft samples were subjected to two digestions in 0.25 M Oxalic acid at 100 °C for 60 min each, followed by centrifugation. Precipitation reagent was added to the supernatant, and the samples were centrifuged. The precipitates were incubated at RT with Fastin Dye Reagent for 90 min at 400 rpm using a thermomixer (Eppendorf, Hamburg, Germany, Cat. No: 5382). The elastin–dye complex was collected by centrifugation and dissolved in Dye Dissociation Reagent before the elastin concentration was measured by absorbance at 513 nm.

#### 4.4.3. Hyaluronic Acid

Levels of HA were quantified using a Purple Jelly Hyaluronan assay kit (Biocolor, Carrickfergus, UK, Cat. No. H2000), according to the manufacturer’s instructions. Briefly, the allografts were digested at 55 °C for 2 h, followed by centrifugation. The supernatant was incubated with precipitation reagent, and the precipitate was resuspended in a solution containing NaCl, followed by incubation with cetylpyridinium chloride (CPC). After centrifugation, the supernatants underwent a second HA precipitation. The samples were then diluted in water to a final concentration that fit within the standard curve range and transferred to a 96-well microplate. Dye reagent was added, incubated for 10 min at RT, and quantified by measuring absorbance at 655 nm.

#### 4.4.4. Proteoglycans

Proteoglycan levels were measured using an sGAG assay kit (Biocolor, Carrickfergus, UK, Cat. No.: B3000) according to the manufacturer’s instructions. The extraction of sGAG was performed using a papain extraction reagent (USP, Rockville, MD, USA, Cat. No. 1495005) at 65 °C for 3 h on a thermoblock (VWR, Radnor, PA, USA, Cat. No.: 76549-938). After incubation, the samples were centrifuged, and blyscan dye reagent was added to the supernatant. The samples were gently mixed and incubated at RT for 30 min at 300 rpm in a thermomixer (Eppendorf, Hamburg, Germany, Cat. No: 5382). Following incubation and two centrifugations, the sGAG precipitates were resuspended in dissociation reagent. The samples were then centrifuged and transferred to a 96-well microplate, and the dye-bound proteoglycan levels were quantified by measuring the absorbance at 656 nm.

### 4.5. Total Protein Extraction and Quantification

The allografts were pulverized at least three times using 1.5 mm zirconium beads (Marshall Scientific, Hampton, NH, USA, D1032-15) in a Beadblaster 24R (Benchmark Scientific, Sayreville, NJ, USA, Cat. No. D2400-R) at 4 °C. The homogenization parameters were as follows: speed at 4260 rpm, duration of 30 s, interruption of 30 s, linear speed of 7 m per second, three cycles, and temperature set at 4 °C. The pulverized samples were suspended in Radioimmunoprecipitation Assay Buffer (RIPA) (Thermo Scientific, Waltham, MA, USA, Cat. No. AAJ63306) supplemented with protease inhibitor cocktail (Thermo Scientific, Cat. No. 78439). The samples were further homogenized in a Beadblaster and incubated overnight at 4 °C. The homogenates were then centrifuged at 13,500 rpm for 20 min at 4 °C. After centrifugation at 4 °C, supernatants containing the total protein extracts were collected, and protein quantification was performed using the Micro Bicinchoninic Acid (microBCA) Assay kit (Thermo Scientific, Cat. No. 23235; TaKaRa, Kusatsu, Japan, Cat. No. T9300A) by measuring absorbance at 450 nm. Protein concentrations were extrapolated from the standard curve, then normalized to graft surface area (μg/cm^2^) and graft weight (μg/mg). Analysis was conducted on samples from eight distinct donors for each CAMP technology, with each sample tested in technical triplicates across four independent experiments.

### 4.6. Growth Factor Levels Analysis

Growth factors levels were quantified utilizing the protein extracts prepared as described in Section 4.5, using commercially available ELISA kits. ANG-2 (Invitrogen, Waltham, MA, USA, Cat. No. KHC1641), EGF (Invitrogen, Cat. No. KHG0061), VEGF (Invitrogen, Cat. No. KHG0111), and PDGF-AA (Invitrogen, Cat. No. EHPDGFA), were obtained from Thermofisher Scientific, and assays were performed following the manufacturer’s protocol provided in each kit. Growth factor concentrations were normalized to the graft’s surface area according to the standard reporting of allograft data [14]. Analysis was conducted on samples from at least six distinct donors for each CAMP technology, with each sample tested in technical triplicates.

### 4.7. Statistical Analysis

Data analysis for ECM quantification and growth factors analyses were conducted using a one-way ANOVA with Tukey’s post-hoc test. A *p* value <0.05 was considered significant. All graphs were expressed as mean ± standard error mean (SEM). Outliers were identified and removed using the ROUT method (Q = 1%). All data were analyzed using GraphPad Prism 10 software.

## 5. Conclusions

This study’s systematic comparison of these distinct layer configurations demonstrates that FT and ACA both retain the IL and chorion and contain a broader range of ECM components and higher concentrations of several key growth factors than Dual Layer. While Dual Layer allografts preserve essential ECM components, they lack the biological diversity observed in FT and ACA. Retaining the IL, and in the case of ACA, an additional amnion layer, results in higher levels of total protein, ECM complexity, and key growth factors. These findings underscore that multi-layer configurations offer an enhanced biochemical profile, potentially translating to enhanced mechanical support and a more favorable wound environment. Although placental allografts are widely used clinically to support wound closure, further research is needed to directly correlate specific layer configurations with clinical outcomes. Overall, this study highlights the novelty and importance of strategic layer retention in optimizing the composition and functionality of placental-derived CAMPs for diverse clinical uses.

## 6. Patents

The subject matter of this manuscript is pending a patent, and IP is owned by Tiger Wound Care.

## Figures and Tables

**Table 1:** Comparison of the total thickness and composition of each layer in three human placental-derived allografts.

	Dual Layer	Full-Thickness	ACA
Total Thickness (μm) ^1^	26.97 ± 16.89	133.23 ± 77.77 ^a^	153.19 ± 57.74 ^a^
Amnion (%) ^2^	100%	4–12%	9–29%
Intermediate Layer (%) ^2^	-	11–33%	7–36%
Chorion (%) ^2^	-	56–86%	38–81%

^1^ Values are represented in mean ± standard deviation (SD), measured in micrometers (μm). ^2^ Values are represented in minimum–maximum percentage of each layer per allograft. ^a^ Statistically significant compared to Dual Layer. Analyzed by one-way ANOVA (*p* < 0.05) with Tukey’s multiple comparison test.

**Table 2:** Levels of growth factors in Dual Layer, Full-Thickness, and ACA.

Growth Factor Levels (pg/cm^2^)	Dual Layer	Full-Thickness	ACA
ANG-2	1.08 × 10^−14^ ± 0	3.05 ± 2.36	27.1 ± 20.9 ^a,b^
EGF	9.12 ± 6.95	33.1 ± 10.9 ^a^	33.9 ± 24.3 ^a^
PDGF-AA	22.3 ± 8.51	179 ± 194	205 ± 97 ^a^
VEGF	20.9 ± 9.24	162 ± 189	196 ± 151

Values are represented in mean ± standard deviation (SD). ^a^ Statistically significant compared to Dual Layer. ^b^ Statistically significant compared to Full Thickness. Analyzed by one-way ANOVA (*p* < 0.05), with Tukey’s multiple comparison test.

## Data Availability

The data presented in this study are available on request from the corresponding author.

## Abbreviations

The following abbreviations are used in this manuscript:

AATB	American Association of Tissue Banks
ACA	Amnion–Intermediate–Chorion–Amnion
ANG-2	Angiopoietin-2
ANOVA	Analysis of Variance
CAMPs	Cellular, Acellular, Matrix-like Products
CRF	Code of Federal Regulation
ECM	Extracellular Matrix
EGF	Epidermal Growth Factor
ELISA	Enzyme-Linked Immunosorbent Assay
FDA	Food and Drug Administration
FT	Full-Thickness
HA	Hyaluronic Acid
HCl	Hydrochloric Acid
hDACM	Dehydrated Human Amnion/Chorion Membrane
H&E	Hematoxylin and Eosin
IL	Intermediate Layer
IND	Investigational New Drug
IP	Intellectual Property
IRB	Institutional Review Board
LLC	Limited Liability Company
MDPI	Multidisciplinary Digital Publishing Institute
microBCA	Micro Bicinchoninic Acid
NaCl	Sodium Chloride
nm	Nanometers
PDGF-AA	Platelet-Derived Growth Factor-AA
RIPA Buffer	Radioimmunoprecipitation Assay Buffer
ROUT	Robust Regression and Outlier Removal
rpm	Rotations Per Minute
RT	Room Temperature
SD	Standard Deviation
SEM	Standard Error Mean
sGAG	Sulfated Glycosaminoglycan
μg/cm^2^	Micrograms per Square Centimeter
μg/mg	Micrograms per Milligram
μm	Micrometer
VEGF	Vascular Endothelial Growth Factor
